# Comparative study on catalytic hydrodehalogenation of halogenated aromatic compounds over Pd/C and Raney Ni catalysts

**DOI:** 10.1038/srep25068

**Published:** 2016-04-26

**Authors:** Xuanxuan Ma, Sujing Liu, Ying Liu, Guodong Gu, Chuanhai Xia

**Affiliations:** 1 School of Resources and Environmental Engineering, Ludong University, Yantai 264025, China; 2 Yantai Institute of Coastal Zone Research, Chinese Academy of Sciences, Yantai 264003, China; 3 Alliance Pharma, Inc. 17 Lee Boulevard Malvern, PA 19355, USA

## Abstract

Catalytic hydrodehalogenation (HDH) has proved to be an efficient approach to dispose halogenated aromatic compounds (HACs). Liquid-phase HDH of single and mixed halobenzenes/4-halophenols with H_2_ over 5% Pd/C and Raney Ni catalyst are investigated and compared. For liquid-phase HDH of single HACs, hydrogenolytic scission reactivity of C-X bonds decreases in order of C-Br > C-Cl > C-I > C-F over Pd/C catalyst, and in order of C-I > C-Br > C-Cl > C-F over Raney Ni catalyst. To clarify the reason why hydrogenolytic scission reactivity of C-X bonds over Pd/C and Raney Ni catalysts exhibits different trends, liquid-phase HDH of mixed HACs over Pd/C and Raney Ni catalysts were studied, and catalysts are characterized by SEM, EDX, and XRD techniques. It was found that the high adsorption of iodoarenes on Pd/C catalyst caused the HDH reactivity of iodoarenes to be lower than that of chloroarenes and bromoarenes in the HDH of single HACs. Moreover, the adsorption of *in situ* produced iodine ion (I^−^) to catalyst surface would result in the decline of catalytic activity, which might be the main reason why the HDH reactivity of HACs in the presence of NaI is rather low.

Halogenated aromatic compounds (HACs) are important and versatile molecules with many applications in synthetic organic chemistry and industrial chemical processes^1^. Due to the existence of halogen atom, many polyhalogenated organic compounds, such as polychlorinated biphenyls (PCBs), polybrominated diphenyl ethers (PBDEs), and perfluorooctane sulfonate (PFOS), exhibit high toxicity, bioaccumulate in the food web, persist in the environment, and pose a risk of causing adverse effects to human health and the environment, and thereby are classified as persistent organic pollutants (POPs)^2^,^3^,^4^,^5^. Therefore, it is urgent to develop an efficient and cost-effective method to detoxify and destroy them with the ever increasing concern about the environment protection. Several methods have been developed for the remediation of HACs, including incineration^6^, microbial degradation^7^, chemical oxidation^8^, photochemical degradation^9^, ultrasonic irradiation^10^, electrolysis^11^, and catalytic hydrodehalogenation (HDH)^12^,^13^,^14^,^15^. Of these methods, catalytic HDH represents a viable alternative and non-destructive treatment for transforming toxic hazardous material into less toxic products that can be more easily degraded or even possess commercial importance^16^,^17^,^18^. Moreover, catalytic HDH of HACs has been accomplished successfully over Rh, Ru, Au, Pt, Pd, and Ni based catalysts^19^,^20^,^21^,^22^,^23^,^24^,^25^,^26^,^27^,^28^.

For the HDH of HACs, the cleavage of carbon-halogen (C-X) bonds is a central matter^29^,^30^,^31^. It is well known that hydrogenolytic scission reactivity of C-X bonds is dependent on C-X bond dissociation energy^29^. On the basis of C-X bond dissociation energy, the HDH reactivity should follow the order, i.e., C-I (222 kJ mol^−1^) > C-Br (280 kJ mol^−1^) > C-Cl (339 kJ mol^−1^) > C-F (456 kJ mol^−1^)^32^. However, there is no agreement on C-X bond cleavage order and key factors influencing the C-X bond cleavage order in catalytic HDH of HACs. Murthy *et al*. studied gas phase HDH of fluorobenzene (FB), chlorobenzene (CB), bromobenzene (BB), and iodobenzene (IB) over Ni/SiO_2_, and found that HDH activity decreased in order of FB > CB > BB > IB, which was attributed to halogen inductive effects^33^. Similarly, Faucher *et al*. found that the reactivity of HACs over Pd/C catalyst decreased in order of CB > BB > IB in liquid phase system, exactly to the true reactivity order of C-X bond^34^. On the other hand, Zinovyev *et al*. investigated liquid-phase HDH of halobenzene mixtures over Pd/C catalyst, and observed that the HDH proceeded consecutively in order of BB, CB, IB, because of the high adsorption of IB to Pd^35^. Also in liquid-phase HDH of FB, CB, BB, and IB using Pd catalyst, Mackenzie and co-workers reported that the reactivity of halobenzenes depended weakly on halogen substituents and was in general order of IB > BB > CB > FB^36^. The reactivity order of halobenzens, IB > BB > CB, is in agreement with the results reported by Abazari *et al*. for liquid-phase HDH of CB, BB, and IB on Pt/Pd/Fe trimetallic nanoparticle^2^. Although these results on HDH reactivity are very useful from academic and practical standpoints, there still is some debate in catalytic HDH reactivity of HACs.

The aim of this work is to study the HDH reactivity of HACs over Pd/C and Raney Ni catalyst in 50% water-ethanol (50/50, v/v). We compared hydrogenolytic scission reactivity of C-X bonds in liquid-phase HDH of single HACs over Pd/C and Raney Ni catalyst with halobenzenes and 4-halophenols as the representative. In order to verify the trend of hydrogenolytic scission reactivity of C-X bonds, liquid-phase HDH of mixed HACs over Pd/C and Raney Ni catalyst were further carried out. Moreover, characterization techniques (SEM, EDX, and XRD) were introduced to elucidate the mechanism for hydrogenolytic scission reactivity of C-X bonds in the HDH of HACs. In addition, the effect of halide ion on catalytic HDH of HACs over Pd/C and Raney Ni catalyst were also investigated.

## Results and Discussion

In our previous work, it was found that *in situ* produced inorganic salt would accumulate on surface of the catalyst in organic solvent, and thus decrease activity of the catalyst in liquid-phase HDH of chlorophenols and PCBs^37^,^38^. Moreover, water in water-organic solvent system could prevent inorganic salt from accumulating on surface of the catalyst and thereby enabled the catalyst to keep high activity in the liquid-phase HDH. Thus, 50% water-ethanol (50/50, v/v) solvent system was applied to liquid-phase HDH of HACs (4-halophenols and halobenzenes) over 5% Pd/C and Raney Ni catalyst here.

**Figure 1:**
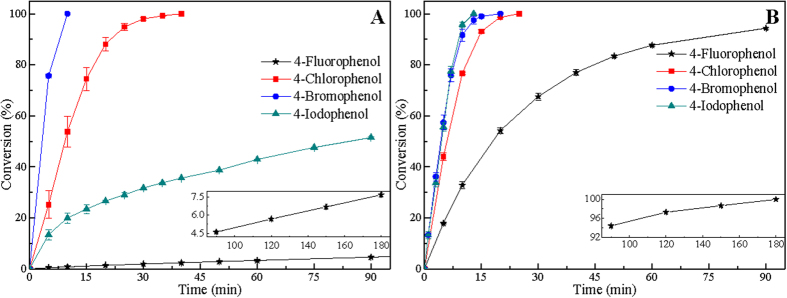
Catalytic HDH of 4-halophenols in ethanol-water (50/50, v/v) over 5% Pd/C (A) and Raney Ni (B) catalysts with reaction time. Reaction conditions: ethanol-water (50/50, v/v) (80 mL), each HAC (1.6 mmol), NaOH (0.0704 g, 1.76 mmol), 5% Pd/C (20 mg), Raney Ni (0.12 g), temperature (30 °C), H_2_: 10 mL min^−1^.

Figure 1 shows the HDH reactivity of 4-FP, 4-CP, 4-BP, and 4-IP over 5% Pd/C and Raney Ni catalyst. For liquid-phase HDH of 4-halophenols, phenol is the final product with no aromatic ring reduction product detected by GC-MS. As is seen in Fig. 1A, 4-CP and 4-BP can be completely hydrodehalogenated using Pd/C catalyst within 40 min and 10 min, respectively. However, the conversion of 4-IP and 4-FP within 90 min are rather low, especially that the conversion of 4-FP is 7.7% within 180 min over Pd/C catalyst. This indicates that Pd/C catalyst exhibits high catalytic activity for the HDH of 4-BP and 4-CP, but shows low catalytic activity for the HDH of 4-IP and 4-FP, especially for the HDH of 4-FP. On the other hand, 4-IP, 4-BP, and 4-CP can be completely hydrodehalogenated over Raney Ni within 13, 20, and 25 min, respectively (Fig. 1B). And what’s more, 4-FP can be hydrodehalogenated completely within 180 min using Raney Ni catalyst under mild condition. These results suggest that Raney Ni not only exhibits high catalytic activity for the HDH of 4-IP, 4-BP, and 4-CP, but is able to effectively catalyze the HDH of 4-FP in 50% water-ethanol (50/50, v/v) under mild conditions (30 °C, 1 atm). In general, carbon-fluorine bonds are among the most passive functionalities in chemistry, and their selective activation and transformation under mild conditions remains a poorly realized challenge^39^. Organofluorines are used widely in numerous applications, but are rather resistant to be hydrodehalogenated under mild condition due to the high stability of the C-F bond^32^,^39^,^40^,^41^. Thus, it is important to develop suitable methods for degradation of organofluorines under mild conditions. And the chemical stability of organofluorines has fueled the search for mild and selective degradation methods^41^,^42^. Here, above results indicate that Raney Ni catalyst exhibits high catalytic activity for the HDH of fluoroarenes and will effectively catalyze C-F bond scission of fluoroarenes under mild conditions. This would provide some guidance to apply Raney Ni catalyst for the HDH of organofluorines, such as PFOS and perfluorooctanoic acid (PFOA).

**Figure 2:**
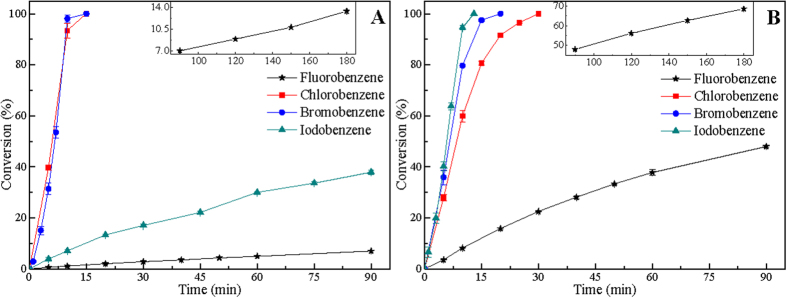
Catalytic HDH of halobenzenes in ethanol-water (50/50, v/v) over 5% Pd/C (A) and Raney Ni (B) catalysts with reaction time. Reaction conditions: ethanol-water (50/50, v/v) (80 mL), each HAC (1.6 mmol), NaOH (0.0704 g, 1.76 mmol), Pd/C (20 mg), Raney Ni (0.12 g), temperature (30 °C), H_2_: 10 mL min^−1^.

Furthermore, it can be seen in Fig. 1A that HDH reactivity of 4-halophenols over Pd/C decreases in order of 4-BP > 4-CP > 4-IP > 4-FP. Yet, for catalytic HDH of 4-halophenols over Raney Ni catalyst, the HDH reactivity decreases in order of 4-IP > 4-BP > 4-CP > 4-FP (Fig. 1B). These results suggest that hydrogenolytic scission reactivity of C-X bonds in the HDH of 4-halophenols over Pd/C catalyst follows the order: C-Br > C-Cl > C-I > C-F. For the HDH of 4-halophenols over Raney Ni, hydrogenolytic scission reactivity of C-X bonds follows the order: C-I > C-Br > C-Cl > C-F. Hence, hydrogenolytic scission reactivity of C-X bonds exhibits different trends in the HDH of 4-halophenols over Pd/C and Raney Ni catalysts. Is the trend for hydrogenolytic scission reactivity of C-X bonds exhibited in the HDH of HACs over Pd/C and Raney Ni catalysts the general trend? Catalytic HDH of halobenzenes over Pd/C and Raney Ni catalyst were carried out, and the corresponding results are presented in Fig. 2. For liquid-phase HDH of halobenzenes, benzene is the final product with no aromatic ring reduction product detected by GC-MS. As shown in Fig. 2A, BB and CB could be hydrodehalogenated completely over Pd/C catalyst within 15 min. However, the conversion of IB and FB are 37.9% and 7.0% within 90 min, respectively. This confirms that Pd/C catalyst exhibits high catalytic activity for hydrogenolytic scission of C-Cl and C-Br bonds in the HDH of CB and BB, but shows low catalytic activity for hydrogenolytic scission of C-I and C-F bonds in the HDH of IB and FB in 50% ethanol-water (50/50, v/v). Meanwhile, it can be seen that IB, BB, and CB are completely hydrodehalogenated over Raney Ni catalyst within 13, 20, and 30 min, respectively (Fig. 2B). For the HDH of FB, the conversions of FB are 48.0% within 90 min and 68.6% within 180 min. Although FB is not completely hydrodehalogenated within 180 min, Raney Ni can still effectively catalyze the HDH of FB under mild conditions (Fig. 2B). These results further indicate that Raney Ni catalyst exhibits high catalytic activity for hydrogenolytic scission of all C-X bonds (C-I, C-Br, C-Cl, and C-F bond) in liquid-phase HDH of halobenzenes under mild conditions. Moreover, the HDH reactivity of halobenzenes over Pd/C and Raney Ni catalysts are in good agreement with the HDH reactivity of 4-halophenols over Pd/C and Raney Ni catalysts, respectively (Figs 1 and 2). These results confirm that hydrogenolytic scission reactivity of C-X bonds in the HDH of HACs decreases in order of C-Br > C-Cl > C-I > C-F over Pd/C catalyst, and in order of C-I > C-Br > C-Cl > C-F over Raney Ni catalyst.

It is widely accepted that the scission reactivity of chemical bond is dependent on the bond dissociation energy^43^. The dissociation energy of C-X bonds decreases in the following order: C-I (222 kJ mol^−1^) < C-Br (280 kJ mol^−1^) < C-Cl (339 kJ mol^−1^) < C-F (456 kJ mol^−1^)^32^. On the basis of C-X bond dissociation energy, the HDH reactivity should follow the order, i.e., C-I > C-Br > C-Cl > C-F^29^. It can be seen in Figs 1B and 2B that hydrogenolytic scission reactivity of C-X bonds in the HDH of HACs over Raney Ni catalyst decreases in order of C-I > C-Br > C-Cl > C-F, which is parallel to the dissociation energy of C-X bonds. Yet hydrogenolytic scission reactivity of C-X bonds in the HDH of HACs over Pd/C catalyst disagrees with the C-X bond dissociation energy. Aramendia *et al*.^44^ reported that liquid-phase HDH reactivity of halobenzenes over palladium supported on AlPO_4_-SiO_2_ catalyst varied in the following sequence: BB > CB > IB > FB. Zinovyev *et al*. also investigated liquid-phase HDH of halobenzenes over Pd/C catalyst, whereby the HDH reactivity decreased in order of BB > CB > IB^35^. In addition, Wu *et al*.^45^ observed the same trend in the HDH of HACs over Ni/C catalyst: BB > CB > IB, which is the same as the HDH reactivity of HACs over Pd/C catalyst but is different from that over Raney Ni catalyst of our experiment. These works on the HDH of HACs indicated that there are still some debates in catalytic HDH reactivity of HACs over Pd/C and Raney Ni catalysts. In order to further investigate the trend for hydrogenolytic scission reactivity of C-X bonds in the HDH of HACs, catalytic HDH of mixed HACs over Pd/C and Raney Ni catalysts were carried out in the following research.

**Figure 3:**
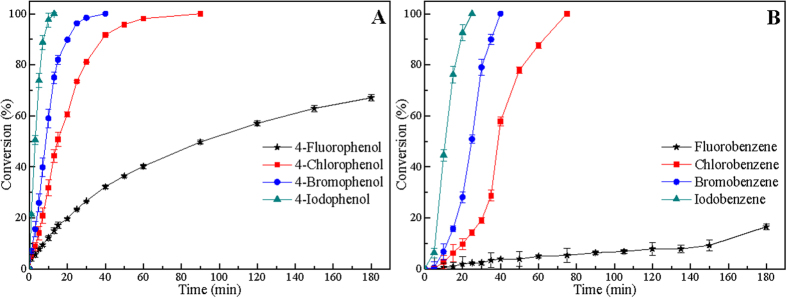
Catalytic HDH of a mixture containing equimolar amounts of 4-halophenols (A) and halobenzenes (B) in ethanol-water (50/50, v/v) over Raney Ni catalyst with reaction time. Reaction conditions: each solvent (80 mL), each HAC (0.8 mmol), NaOH (0.105 g, 3.52 mmol), Raney Ni (0.12 g), temperature (30 °C), H_2_: 10 mL min^−1^.

**Figure 4:**
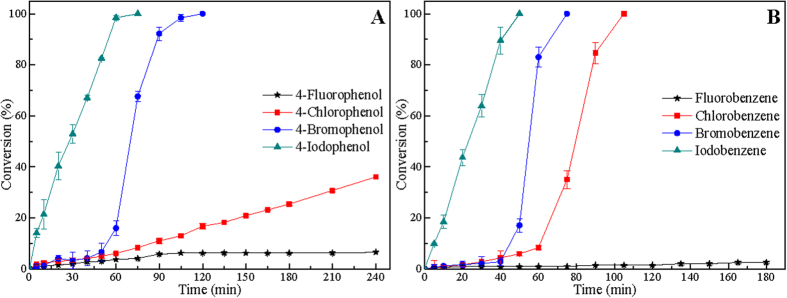
Catalytic HDH of a mixture containing equimolar amounts of of 4-halophenols (A) and halobenzenes (B) in ethanol-water (50/50, v/v) over 5% Pd/C catalyst with reaction time. Reaction conditions: each solvent (80 mL), each HAC (0.8 mmol), NaOH (0.105 g, 3.52 mmol), 5% Pd/C (20 mg), temperature (30 °C), H_2_: 10 mL min^−1^.

Figure 3 shows the results for liquid-phase HDH of mixed 4-halophenols/halobenzenes over Raney Ni catalyst. For liquid-phase HDH of mixed 4-halophenols, phenol is the sole product. As can be seen in Fig. 3A, the HDH reactivity of mixed 4-halophenols over Raney Ni catalyst follows the order: 4-IP > 4-BP > 4-CP > 4-FP. Similarly, the HDH reactivity of mixed halobenzenes over Raney Ni catalyst are in general order of IB > BB > BB > FB, which is in agreement with the HDH of halobenzenes over Raney Ni catalyst and obeys the order of the C-X bond dissociation energy. Subsequently, catalytic HDH of a mixture of halobenzenes/4-halophenols with hydrogen over 5% Pd/C catalyst was further investigated (Fig. 4). As illustrated in Fig. 4A, 4-IP is completely hydrodehalogenated over Pd/C catalyst first, follows by 4-BP, 4-CP, and 4-FP. Similarly, the HDH order of mixed halobenzenes over Pd/C catalyst follows the order: IB > BB > CB > FB. Above results indicate that the HDH reactivity of mixed HACs disagrees with the HDH reactivity of single HACs over Pd/C catalyst, but is consistent with the sequence of C-X bonds strength. In short, HACs with lower C-X bond strength will be hydrodehalogenated much easier over Pd/C catalyst.

For liquid-phase HDH of HACs over Pd/C, HDH of single and mixed HACs exhibit different trends. Whether hydrogenolytic scission reactivity of C-X bonds in the HDH of HACs obeys the order of C-X bond dissociation energy or not? It is noteworthy that halobenzenes are hydrodehalogenated with a surprising “consecutive” selectivity; i.e., the HDH of IB selectively precedes that of BB, CB, and FB (Fig. 4A). Also, catalytic HDH of mixed 4-halophenols is highly selective; 4-IP reacts first, followed by 4-BP, 4-CP, and 4-FP (Fig. 4B). In both cases, only when IB or 4-IP almost converts to benzene/phenol, the other HACs can be hydrodehalogenated, which indicates that the presence of iodoarenes inhibits the HDH of bromoarenes, chloroarenes, and fluoroarenes to some extent. In the literature reported previously, the adsorption of HACs on Pd/C catalyst has been determined in the decreasing order: iodoarenes> bromoarenes > chloroarenes > fluoroarenes^35^,^46^. In the HDH of mixed HACs over Pd/C catalyst, iodoarene was hydrodehalogenated preferentially might be due to the high adsorption of iodoarene on the catalyst^36^.

To ascertain whether the selective order of HDH reactivity of HACs could be changed by an excess of catalyst, catalytic HDH of a mixture of 4-halophenols with an excess of 5% Pd/C catalyst (100 mg) was studied (Figure S1). As shown in Figure S1, the HDH reactivity of 4-halophenols is obviously improved as the amount of Pd/C catalyst increases from 20 mg to 100 mg. However, similar sequence of profiles is observed for the HDH of mixed 4-halophenols over an excess of 5% Pd/C catalyst. These results confirm that the selective order for the HDH of mixed HACs over Pd/C catalyst is mainly due to the high adsorption of iodoarene on the catalyst. On the other hand, we compared the HDH reactivity of mixed HACs and single HACs over Pd/C and Raney Ni catalyst (Table S1). As presented in Table S1, conversion rates of fluoroarenes, chloroarenes, and bromoarenes in HACs mixtures are slower than that of single fluoroarenes, chloroarenes, and bromoarenes over Pd/C and Raney Ni catalysts. This implies that the HDH reactivity of fluoroarenes, chloroarenes, and bromoarenes in HACs mixtures is obviously delayed. When iodoarene is completely hydrodehalogenated, there shall be no iodoarene adsorbed on the catalyst. Hence, there might be some other factors affecting the HDH reactivity of HACs over Pd/C and Raney Ni catalysts.

In an attempt to clarify the reason for the delayed effects in the HDH of mixed HACs, catalyst characterizations (SEM, EDX, and XRD) were introduced to analyze catalyst samples before and after the HDH in 50% water-ethanol (50/50, v/v).

**Figure 5:**
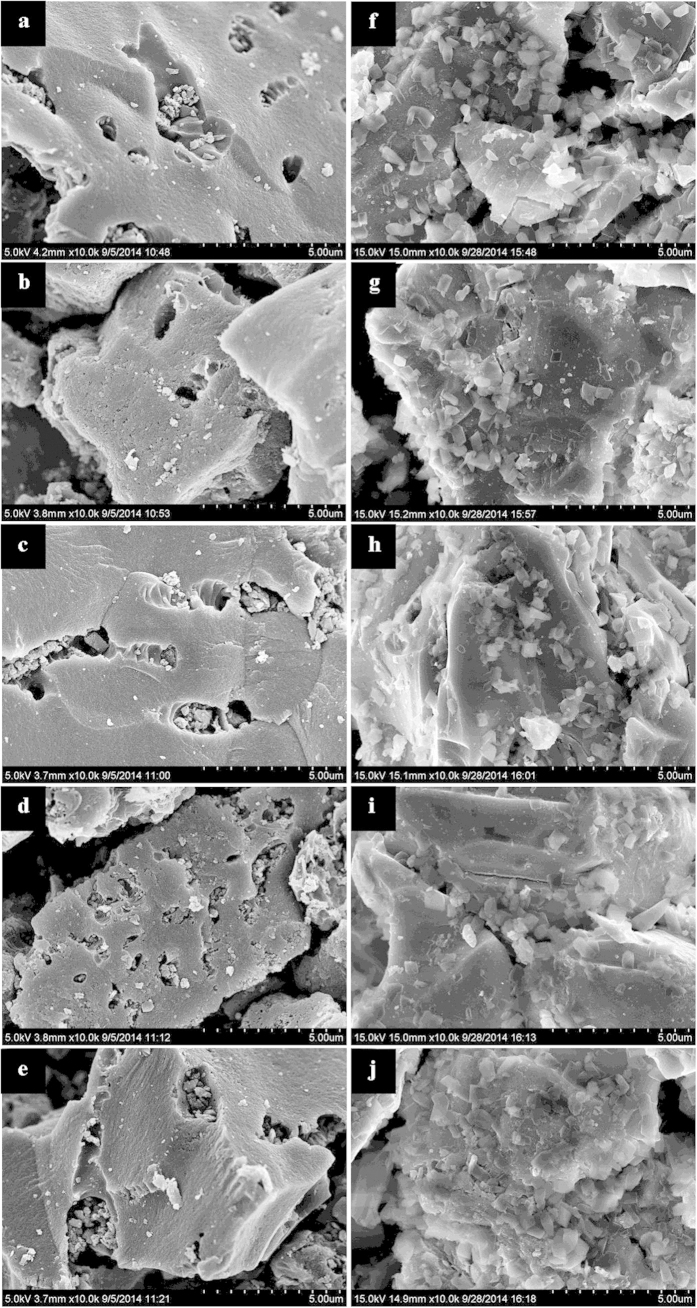
Representative SEM images of (**a**) fresh 5% Pd/C catalyst, (**b**) 5% Pd/C catalyst used in the HDH of fluoroarene, (**c**) 5% Pd/C catalyst used in the HDH of chloroarene, (**d**) 5% Pd/C catalyst used in the HDH of bromoarene, (**e**) 5% Pd/C catalyst used in the HDH of iodoarene, (**f**) fresh Raney Ni catalyst, (**g**) Raney Ni catalyst used in the HDH of fluoroarene, (**h**) Raney Ni catalyst used in the HDH of chloroarene, (**i**) Raney Ni catalyst used in the HDH of bromoarene, (**j**) Raney Ni catalyst used in the HDH of iodoarene.

**Figure 6:**
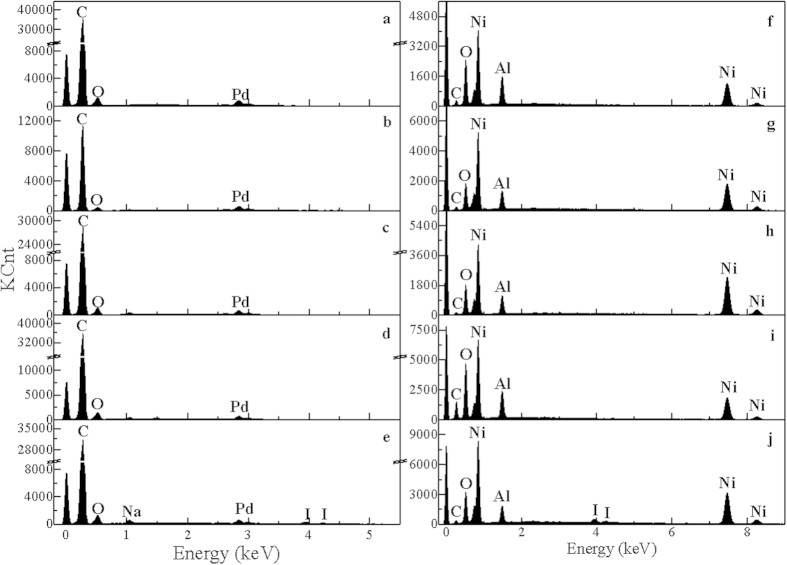
EDX spectra of (**a**) fresh 5% Pd/C catalyst, (**b**) 5% Pd/C catalyst used in the HDH of fluoroarene, (**c**) 5% Pd/C catalyst used in the HDH of chloroarene, (**d**) 5% Pd/C catalyst used in the HDH of bromoarene, (**e**) 5% Pd/C catalyst used in the HDH of iodoarene, (**f**) fresh Raney Ni catalyst, (**g**) Raney Ni catalyst used in the HDH of fluoroarene, (**h**) Raney Ni catalyst used in the HDH of chloroarene, (**i**) Raney Ni catalyst used in the HDH of bromoarene, (**j**) Raney Ni catalyst used in the HDH of iodoarene.

Figure 5 shows the representative SEM images of Pd/C and Raney Ni catalysts before and after liquid-phase HDH. As illustrated in Fig. 5a–e, the surface morphology of Pd/C catalysts used in 50% water-ethanol (50/50, v/v) are almost the same as that of the fresh catalyst. Similar phenomena are observed for the representative SEM images of Raney Ni catalysts before and after liquid-phase HDH (Fig. 5f–j). Subsequently, SEM-EDX analyses of the catalysts were performed to obtain information about elemental composition of the catalyst surface (Fig. 6). For Pd/C catalysts before and after liquid-phase HDH, the characteristic peaks of Pd and C are present in EDX spectra of all the catalysts, which reveals that elements of Pd and C exist in all the catalysts. As shown in Fig. 6, there is no other peak in the catalyst samples used in the HDH of fluoroarene, chloroarene, and bromoarene, indicating that the elemental composition of the catalyst surface does not change. Yet there are additional peaks for elements of Na and I in catalyst samples used in the HDH of iodoarene. Similar results were observed for Raney Ni catalysts before and after liquid-phase HDH. These results indicate that the elemental composition of catalyst surface is different before and after the HDH of iodoarene for Pd/C and Raney Ni catalysts.

**Figure 7:**
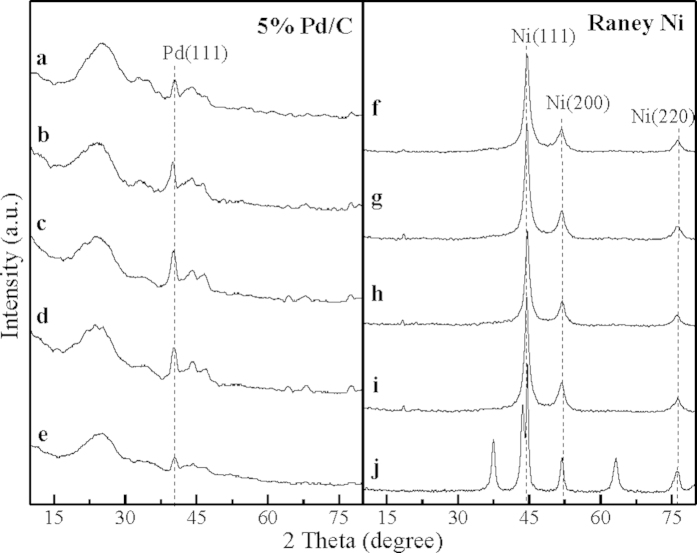
XRD pattern of (**a**) fresh 5% Pd/C catalyst, (**b**) 5% Pd/C catalyst used in the HDH of fluoroarene, (**c**) 5% Pd/C catalyst used in the HDH of chloroarene, (**d**) 5% Pd/C catalyst used in the HDH of bromoarene, (**e**) 5% Pd/C catalyst used in the HDH of iodoarene, (**f**) fresh Raney Ni catalyst, (**g**) Raney Ni catalyst used in the HDH of fluoroarene, (**h**) Raney Ni catalyst used in the HDH of chloroarene, (**i**) Raney Ni catalyst used in the HDH of bromoarene, (**j**) Raney Ni catalyst used in the HDH of iodoarene.

To obtain further information about the catalyst surface composition, XRD analysis was performed for Pd/C and Raney Ni catalysts before and after liquid-phase HDH (Fig. 7). For the XRD pattern of 5% Pd/C catalysts before and after the HDH (Fig. 7a–e), the peak centers at about 40.1° is indexed to (1 1 1) Pd plane^2^. Except for peak of Pd, no peak is found in the XRD patterns of 5% Pd/C catalysts used in the HDH of HACs. However, compared with fresh Pd/C catalyst, the characteristic peaks of (1 1 1) Pd is weakened for Pd/C catalyst used in the HDH of iodoarene. This might be due to the adsorption of I^−^ on the surface of Pd/C catalyst. On the other hand, for the XRD patterns of Raney Ni catalysts, the peaks at 2θ = 44.5°, 51.8°, and 76.3° can be indexed to (1 1 1), (2 0 0), and (2 2 0) planes of metallic nickel, respectively^47^. Compared with the XRD pattern of fresh Raney Ni catalyst, there are three more peaks at 2θ = 37.5°, 43.6°, and 63.2° in the XRD pattern of Raney Ni catalysts used in the HDH of iodoarene. According to JCPDS (Joint Committee on Powder Diffraction Standards) standard card, the three peaks are identified to (2 2 0), (3 1 1), and (4 2 0) NaI planes, respectively. These results confirm that some substrates adsorb on the surface of Raney Ni catalysts used in the HDH of iodoarene and these substrates are NaI crystals. However, the element of I on Pd/C and Raney Ni catalyst exists in different forms.

In liquid-phase HDH, HX is generated as byproduct and would poison the catalytically active metal^29^. And base is always used to serve as proton scavenger, eliminating the negative effect of HX on the catalyst^48^. In this paper, NaOH was added to prevent catalyst deactivation caused by HX, and sodium halide (NaX) was the product of neutralization of HX and NaOH^49^. In fact, water in 50% ethanol-water (v/v, 50/50) could prevent inorganic salts from depositing on the surface of catalyst, and enabled the catalyst to keep high activity and stability in liquid-phase hydrodechlorination of chloroarenes^37^,^38^. Thus, it is reasonable that no NaF, NaCl, or NaBr deposited on the surface of Pd/C and Raney Ni catalysts according to characterization analyses (SEM, EDX, and XRD). However, the characterization analyses suggest that I^−^, produced *in situ*, would adsorb on the catalyst surface because of its high adsorption. This may be the main reason why the HDH reactivity of mixed HACs is lower than that of single HACs.

According to above catalyst characterization and HDH of single and mixed HACs, it will be reasonable to presume that I^−^, produced *in situ*, may adsorb on the catalyst surface and thus inhibit liquid-phase HDH of HACs over Raney Ni and Pd/C catalyst. Therefore, additional experiments were conducted to study the effect of halide ion (X^−^) on the HDH of HACs over Pd/C and Raney Ni catalysts. Liquid-phase HDH of 4-halophenols (4-CP, 4-BP, and 4-IP) over Pd/C catalyst without additive was compared to HDH of 4-halophenols with addition of 1.0 mmol sodium halide. As shown in Table 1 (entries 1–15), the presence of F^−^, Cl^−^, and Br^−^ have little effect on the HDH reactivity of 4-CP, 4-BP, and 4-IP over Pd/C catalyst. Yet, when 1.0 mmol I^−^ is present in reaction system, the HDH reactivity of 4-CP, 4-BP, and 4-IP are reduced by factors of 7.1, 4.9, and 1.3, respectively. This indicates that I^−^ has a strong inhibitory effect on Pd-based catalysts, which might be attributed to its strong adsorption to metallic surfaces^36^. Further, the effect of X^−^ on the HDH of HACs over Raney Ni catalyst was examined (entries 16–30, Table 1). It can be observed in Table 1 that F^−^, Cl^−^, and Br^−^ have no effect on conversion rate of 4-CP, 4-BP, and 4-IP using Raney Ni as catalyst. Nevertheless, the HDH reactivity of 4-CP, 4-BP, and 4-IP are reduced by factors of 2.3, 1.1, and 1.2 in the presence of 1 mmol I^−^. The decreased HDH reactivity of 4-CP, 4-BP, and 4-IP may be caused by the adsorption of I^−^ on Raney Ni catalyst. Overall, I^−^, produced *in situ*, will adsorb on the catalyst surface and thus inhibit liquid-phase HDH of HACs over Raney Ni and Pd/C catalysts. This may explain why conversion rates of fluoroarenes, chloroarenes, and bromoarenes in HACs mixtures are slower than that of single fluoroarenes, chloroarenes, and bromoarenes over Pd/C and Raney Ni catalysts (Table S1). Moreover, the influence of I^−^ on Pd/C catalyst is stronger than that of I^−^ on Raney Ni catalyst.

As F^−^, Cl^−^, and Br^−^ have little effect on Pd/C catalyst in liquid-phase of HACs in 50% water-ethanol (50/50, v/v), the catalyst exhibits high catalytic activity, and thus the HDH reactivity of chloroarene and bromoarene are much higher than that of iodoarene (Figs 1 and 2). For the HDH of HACs over Pd/C catalyst, the HDH reactivity of iodobenzene and 4-iodophenol are very poor mainly due to the decrease of catalytic activity caused by the high adsorption of iodoarenes and *in situ* produced I^−^. On the other hand, for catalytic HDH of mixed HACs over Pd/C catalyst, iodoarene reacts first, follows by bromoarene, chloroarene, and fluoroarene (Figs 3 and 4) because of the low C-I bond dissociation energy and high adsorption of iodoarenes on the catalyst. It is believed that hydrogenolytic scission reactivity of C-X bond for liquid-phase HDH of HACs over Pd/C catalyst still obeys the order of C-X bond dissociation energy, i.e., C-I > C-Br > C-Cl > C-F. However, the adsorption of iodoarenes and *in situ* produced I^−^ have considerable effect on liquid-phase HDH of HACs.

## Conclusions

For liquid-phase HDH of single HACs, hydrogenolytic scission reactivity of C-X bonds decreases in order of C-Br > C-Cl > C-I > C-F over Pd/C catalyst, and in order of C-I > C-Br > C-Cl > C-F over Raney Ni catalyst. For liquid-phase HDH of mixed HACs over Pd/C and Raney Ni catalysts, hydrogenolytic scission reactivity of C-X bonds decreases in order of C-I > C-Br > C-Cl > C-F. Based on comparison between the HDH reactivity of single and mixed HACs over Pd/C catalyst, it will be reasonable to presume that the HDH reactivity of iodoarenes is lower than that of chloroarenes and bromoarenes in the HDH of single HACs due to the high adsorption of iodoarenes on the catalyst.

For liquid-phase HDH of single HACs, Pd/C catalyst exhibits high catalytic activity for the HDH of bromoarenes and chloroarenes, but shows low catalytic activity for the HDH of iodoarenes and fluoroarenes. According to catalyst characterization (SEM, EDX, and XRD), I^−^, produced *in situ*, will adsorb on the catalyst surface. The high adsorption of *in situ* produced I^−^ can lead to the decrease of the catalytic activity in liquid-phase HDH of HACs, and thereby the HDH reactivity of mixed HACs is lower than that of the HDH reactivity of single HACs. This may be the main reason why the HDH reactivity of HACs in the presence of NaI is rather low.

For liquid-phase HDH of HACs, Raney Ni not only exhibits high catalytic activity for the HDH of iodoarenes, bromoarenes, and chloroarenes, but can effectively catalyze the HDH of fluoroarenes under mild conditions (30 °C, 1 atm). This will provide some significant guidance to develop some Ni-based catalyst for the HDH of polyfluorinated hydrocarbons (such as PFOS and PFOA).

## Methods

### Chemicals

Raney Ni (RTH-3110) and 5% Pd/C catalyst used in this study were purchased from Dalian Tongyong Chemical Co., Ltd., Liaoning, China. The weight percentage of nickel and aluminum in the catalyst were more than 90% and less than 7%, respectively. Raney Ni and Pd/C catalyst are not pre-treated before all experiments, and they were kept in water-sealing storage and hermetical desicator, respectively.

All HACs including fluorobenzene (FB), chlorobenzene (CB), bromobenzene (BB), iodobenzene (IB), 4-fluorophenol (4-FP), 4-chlorophenol (4-CP), 4-bromophenol (4-BP), and 4-iodophenol (4-IP) used in experiments were purchased from Sigma-Aldrich with a minimum purity of 98%. The other reagents such as EtOH, NaOH, NaF, NaCl, NaBr, and NaI are analytical grade and supplied by Sinopharm Chemical Reagent Co., Ltd., Shanghai, China. Deionized water was used in the reaction. The purity of hydrogen and nitrogen used in the experiments is more than 99.99%.

### Catalytic procedure

All the liquid-phase HDH reactions were carried out in a three-neck flask, which was attached with a thermometer, a condenser and a hydrotreater (including a hydrogen cylinder, hydrogen flowmeter, three-way valve and a nitrogen cylinder), with a magnetic stirrer. The reaction vessel (100 mL) was placed in a temperature-controlled heating water bath with a precision of ±0.5 °C. At the beginning of each experiment, 80 mL solution was added into the flask, containing HAC and NaOH. After the air in the flask was completely replaced by nitrogen, Raney Ni or 5% Pd/C catalyst was added and agitation was started.

### Analytical methods

The intermediate products in the HDH of HACs were determined by GC/MS (Thermo Scientific ITQ 900) with a column of TR-5MS (30 m × 0.25 mm × 0.25 *μ*m). The composition of the reaction/product mixture was analyzed by gas chromatography (Agilent 7890A), employing a flame ionization detector (FID) and a column of DB-1701 (30 m × 0.32 mm × 0.25 *μ*m). Prior to analysis, the basic solution samples were neutralized with dilute CH_3_COOH (ca. 0.2 mol L^−1^). The detection limit of GC analysis for HACs was 1 pg mL^−1^. Taking 4-chlorophenol as a representative reactant, the conversion is defined as:


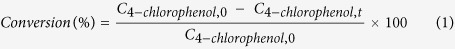


where C_*4-chlorophenol, 0*_ and C_*4-chlorophenol, t*_ represent the initial concentration of 4-chlorophenol and the value at time *t*, respectively. And conversion rate is given as:


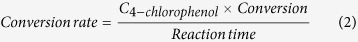


where *C*_*4-chlorophenol*_ represents total amount of substance of 4-chlorophenol. The conversion rate is the average one within reaction time.

### Characterizations

The catalysts after HDH reaction in liquid-phase system were separated from the solution, washed with 95% ethanol, isopropanol, and *n*-hexane to remove adsorbed organic compounds, and dried under N_2_ flow at 200 °C prior to characterization analysis^37^,^38^. The surface morphology of fresh and used catalysts were characterized using a Hitachi S-4800 field emission scanning electron microscope (FE-SEM) coupled with an energy dispersive X-ray spectrometer (EDXS, HORIBA EMAX Energy EX-350) used for chemical elemental analysis. X-ray diffractograms (XRD) patterns of the catalysts were recorded with a Shimadzu XRD-6100 using nickel filtered Cu K*α* radiation. The samples were scanned at a rate of 0.1°/s over the 5° ≤ 2θ ≤ 80° range with a scan time of 5 s step^−1^.

## Additional Information

**How to cite this article**: Ma, X. *et al*. Comparative study on catalytic hydrodehalogenation of halogenated aromatic compounds over Pd/C and Raney Ni catalysts. *Sci. Rep*. **6**, 25068; doi: 10.1038/srep25068 (2016).

## Supplementary Material

Supplementary Information

## Figures and Tables

**Table 1:** The effect of halide anions on liquid-phase HDH of HACs over 5% Pd/C and Raney Ni catalysts in ethanol-water (50/50, v/v).

Entry	Catalyst	HAC^a^	halide anion	Time (min)	Conversion (%)^b^	Conversion rate (mmol L^−1^ min^−1^)	factor^c^
1	Pd/C	4-CP	no	40	100	0.500	–
2			F^−^	40	100	0.500	1.0
3			Cl^−^	40	100	0.500	1.0
4			Br^−^	40	100	0.500	1.0
5			I^−^	40	13.9	0.070	7.1
6		4-BP	no	10	100	2.000	–
7			F^−^	10	100	2.000	1.0
8			Cl^−^	10	100	2.000	1.0
9			Br^−^	10	100	2.000	1.0
10			I^−^	10	97.3	0.408	4.9
11		4-IP	no	90	57.8	0.128	–
12			F^−^	90	56.7	0.126	1.0
13			Cl^−^	90	57.9	0.129	1.0
14			Br^−^	90	57.5	0.128	1.0
15			I^−^	90	44.2	0.098	1.3
16	Raney Ni	4-CP	no	25	100	0.800	–
17			F^−^	25	99.1	0.793	1.0
18			Cl^−^	25	99.2	0.794	1.0
19			Br^−^	25	99.2	0.794	1.0
20			I^−^	25	86.5	0.346	2.3
21		4-BP	no	20	100	1.000	–
22			F^−^	20	100	1.000	1.0
23			Cl^−^	20	100	1.000	1.0
24			Br^−^	20	100	1.000	1.0
25			I^−^	20	95.3	0.953	1.1
26		4-IP	no	15	100	1.333	–
27			F^−^	15	100	1.333	1.0
28			Cl^−^	15	100	1.333	1.0
29			Br^−^	15	100	1.333	1.0
30			I^−^	15	80.8	1.077	1.2

^a^Reaction conditions: solvent (80 mL), each HAC (1.6 mmol), NaOH (0.0704 g, 1.76 mmol), halide anion (1.0 mmol), 5% Pd/C (20 mg), Raney Ni (0.12 g), temperature (30 °C), H_2_: 10 mL min^−1^.

^b^Products and yields were determined by GC-MS and GC-FID.

^c^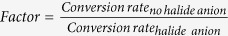.
