# Supplementary material for: Comparative study on catalytic hydrodehalogenation of halogenated aromatic compounds over Pd/C and Raney Ni catalysts
Source: Sci Rep. 2016 Apr 26;6:25068. doi: 10.1038/srep25068 (PMC4844995; doi:10.1038/srep25068)
Supplement: Supplementary Information [file srep25068-s1.pdf]

Comparative study on catalytic hydrodehalogenation of halogenated aromatic compounds over Pd/C and Raney Ni catalyst

Xuanxuan Ma[1], Sujing Liu[2], Ying Liu[1, *], Guodong Gu[3], Chuanhai Xia[1, *]

[1] School of Resources and Environmental Engineering, Ludong University, Yantai 264025, China

[2] Yantai Institute of Coastal Zone Research, Chinese Academy of Sciences, Yantai 264003, China

[3] Alliance Pharma, Inc. 17 Lee Boulevard Malvern, PA 19355, USA

* Corresponding author:

Dr. Ying Liu, E-mail: liuyingldu@hotmail.com

Prof. Dr. Chuanhai Xia, E-mail: chxia_ldu@hotmail.com

Tel.: +86 535 6016605

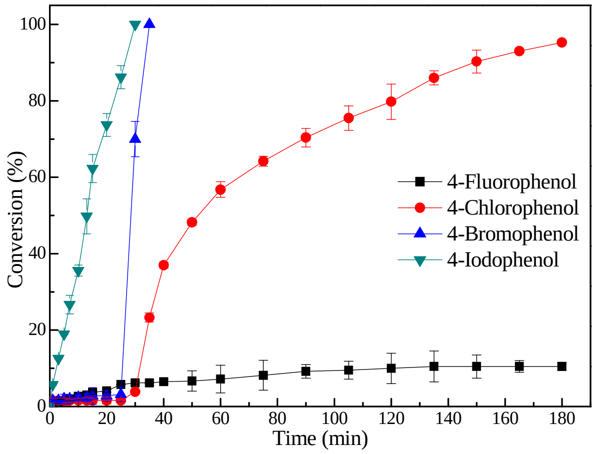

Figure S1. Catalytic HDH of a mixture containing equimolar amounts of 4-halophenols in ethanol-water (50/50, v/v) over 100 mg 5% Pd/C catalyst with reaction time. Reaction conditions: each solvent (80 mL), each HACs (0.8 mmol), NaOH (0.105 g, 3.52 mmol), 5% Pd/C (100 mg), temperature (30 ℃), H_2: 10 mL min^{-1}.

Table S1. Liquid-phase HDH reactivity of single and mixed HACs over 5% Pd/C and Raney Ni catalysts in ethanol-water (50/50, v/v)

Entry	Catalyst	HAC[a]	Conversion rate (mol L^{-1} min^{-1})[b]	
			Single	Mixed
1	5% Pd/C	4-FP	0.009	0.003 (0-240 min)
2		4-CP	0.500	0.015 (0-240 min)
3		4-BP	2.000	0.143 (50-120 min)
4		4-IP	0.114	0.133 (0-75 min)
5		FB	0.015	0.001 (0-180 min)
6		CB	1.333	0.222 (60-105 min)
7		BB	1.333	0.286 (40-75 min)
8		IB	0.084	0.200 (0-50 min)
9	Raney Ni	4-FP	0.111	0.038 (0-180 min)
10		4-CP	0.800	0.111 (0-90 min)
11		4-BP	1.000	0.250 (0-40 min)
12		4-IP	1.538	0.769 (0-13 min)
13		FB	0.076	0.009 (0-180 min)
14		CB	0.667	0.133 (0-75 min)
15		BB	1.000	0.250 (0-40 min)
16		IB	1.333	0.400 (0-25 min)

[a] Reaction conditions: solvent (80 mL), 5% Pd/C (20 mg), Raney Ni (0.12 g), temperature (30 ℃), H_2: 10 mL min^{-1}.

[b] Products and yields were determined by GC-MS and GC-FID.
